# Muscle dysmorphia subscale of eating disorder assessment for men: validity and reliability of the Portuguese version in men across multiple ages

**DOI:** 10.1192/j.eurpsy.2024.1172

**Published:** 2024-08-27

**Authors:** A. Silva, A. Macedo, M. J. Brito, C. Marques, A. T. Pereira, M. Bajouco

**Affiliations:** ^1^Faculty of Medicine, University of Coimbra, Coimbra, Portugal; ^2^Institute of Psychological Medicine, Faculty of Medicine, University of Coimbra, Coimbra, Portugal

## Abstract

**Introduction:**

Although symptom presentation varies by gender, almost all eating disorder/ED instruments have been developed and validated on females. The Eating Disorder Assessment for Men (EDAM; Stanford & Lemberg 2012) is a male specific self-report measure, composed of four sub-scales, proved to be useful to assess gender differences in ED presentations (Nagata et al. 2021). The MD comprises 5 items about the overwhelming concern with muscularity and the false perception of having an underdeveloped body.

**Objectives:**

Having already valid measures of body image and eating behaviors in men, we now aim to analyze the psychometric properties of the Portuguese version of MD, in order to have a quick and rigorous measure of this specific construct.

**Methods:**

Participants were 227 male individuals (mean age=30.41 years±13.96; range: 14-73 years). They answered an online survey including the preliminary DM and the Portuguese validated versions of the Eating Disorder Examination Questionnaire (EDE-Q7) and the Body Image Concern Inventory (BICI).

**Results:**

Confirmatory Factor Analysis showed that the unidimensional model presented good fit indexes (χ^2^/df=.6829; RMSEA=.0000; CFI=1.00 TLI=1.01, GFI=.995). Cronbach’s alfa was .891; all the items contributed to the internal consistency and had high internal validity. Pearson correlations of DM with EDE-Q7 and BICI were significant (p<.001) and moderate-high, respectively,.384 and .522.

**Conclusions:**

The Portuguese preliminary version of DM-EDAM demonstrated validity (construct and convergent) and reliability. can be used for clinical and research purposes, namely in an ongoing project we have in progress, about body image, disordered eating, gender and age.

**Disclosure of Interest:**

None Declared

